# Demography of grandmothering: a case study in Agta foragers

**DOI:** 10.1098/rspb.2025.0385

**Published:** 2025-05-14

**Authors:** Abigail E. Page, Mark Dyble, Andrea Migliano, Nikhil Chaudhary, Sylvain Viguier, Daniel Major-Smith

**Affiliations:** ^1^ Division of Psychology, Brunel University London, Uxbridge Campus, London, UK; ^2^ Department of Archaeology, University of Cambridge, Cambridge, UK; ^3^ Department of Anthropology, University of Zurich, Zurich, Switzerland; ^4^ Graphcore, London, UK; ^5^ Population Health Sciences, Bristol Medical School, University of Bristol, Bristol, UK; ^6^ Department for the Study of Religion, School of Culture and Society, Aarhus Universitet, Aarhus, Central Denmark Region, Denmark

**Keywords:** demography, grandmothering, Agta, post-reproductive lifespan, hunter–gatherers, allomothering

## Abstract

Grandmothers are often presented as key carers due to low costs and high inclusive fitness returns. Empirically, however, grandmothers are not consistently important. Understanding the factors that promote, or hinder, grandmothering is an important next step. We explore the demographic predictors of the low levels of grandmothering in Agta hunter–gatherers (78 children with 29 grandmothers). Due to generational reproductive timing, grandmothers still had dependent children until, on average 52, creating reproductive overlap. The minimal levels of grandmaternal investment after the age of 60 are explained by declining health and high mortality. This means the ‘helping window’ for grandmothering only spans 7 years. Yet grandmothers are still limited by multiple dependent grandchildren in this period, given high fertility. We suggest then that Agta grandmothering is constrained by (i) reproductive overlap and (ii) grandchildren competition. Accordingly, we tested how (i) the number of children and (ii) grandchildren associated with grandmothering using Bayesian mixed-effect models. We found moderate to strong evidence that more children/grandchildren reduced investment in each grandchild. Consequently, whether Agta grandmothers help appears dependent on demographic schedules, which vary widely both within and between populations. Future formal demographic modelling will then help shed light on the evolution of grandmothering in humans.

## 1. Introduction

Across time, cultures, economic systems and socioeconomic classes, women have been assisted by a range of allomothers (non-maternal individuals who invest in the mother and child), including kin and non-kin [1–6]. Allomothering is broadly defined as practical support directed at the child (childcare), the wider household (domestic tasks and resource production) and emotional and informational support to the mother [1,7–10]. Among the myriad of allomothers available to mothers, particular attention has been paid to grandmothers, with Hrdy [11] labelling them as an ‘ace in the hole’ [10]. From an inclusive fitness framework, grandmothers have high levels of relatedness with their grandchildren, low costs of allomaternal investment and have the knowledge and skills required to ensure children’s survival and well-being [11]. Of all possible allomothers, grandmothers have been most commonly associated with positive outcomes for the mother and child [5,11–22]. As a result of these observations, the ‘grandmothering hypothesis’ posits that the women’s post-reproductive lifespan (which is significantly longer in humans than in other species, including other great apes [23]) evolved due to the inclusive fitness advantages acquired by investing in their grandchildren [10,24,25].

The empirical evidence, however, does not consistently demonstrate that grandmothers are (i) reliably associated with improved mother and/or grandchild outcomes *or* (ii) consistent allomothers in terms of investment. For instance, one global review highlighted a lack of clear patterns between grandmaternal investment and child outcomes, stressing the importance of context-dependent outcomes [26]. Other studies have demonstrated that in matrilineal societies, maternal grandmothers are associated with higher child mortality due to resource competition [27], and many studies report null effects [21]. In particular, paternal grandmothers have been documented to rarely have positive associations with child outcomes, and are more frequently associated with poorer outcomes [21,22,28–30]. Observational studies of grandmaternal investment also indicate that, in some contexts, grandmothers have little involvement in direct childcare [31,32] and provisioning [9]. Reflecting on the Aché and Hiwi (South American foragers) grandmothers’ low levels of provisioning, Hill & Hurtado [9] demonstrate that, given high mobility and mortality rates, only around 10% of women in their 30s co-resided with their mothers. This appreciably decreases the ability of mothers to rely on grandmaternal support, calling into question the universality of grandmothering. This then poses the question: what may be systematically different about some populations that restricts—or, conversely, promotes—grandmothering? Answering this question will help us understand the diversity in grandmothering, and thus better understand its evolution. While we cannot claim to answer this question here, we use Agta hunter–gatherers as a useful case study to present and highlight these issues in one society, which can help inform future cross-cultural work.

### (a) Allomaternal childcare in the Agta

Our previous research on allomothering in the Agta [33–36] paints a similar picture of grandmaternal childcare to other horticultural and hunter–gatherer societies [3,37]. Childcare networks are diverse, comprising kin and non-kin, as well as juveniles (in the form of mixed age and sex playgroups), reproductive and post-reproductive adults. However, from the child’s perspective (i.e. the proportional representation of childcare from one source), grandmothers do not represent a major source of direct support (electronic supplementary material, figures S1–S4). In infancy (0−2 years), children on average received 3% of their total childcare investments from their maternal grandmother and 0.3% from paternal grandmothers. Toddlers (2–5 years) received 1.9% of their total childcare investments from their maternal grandmother and 0.3% from their paternal grandmother. Among the 78 children, only 28% and 10% received *any* direct childcare from their maternal and paternal grandmothers, respectively, since many were not co-resident (32% and 14% co-resided with a maternal and paternal grandmother, respectively). This is both a product of high mobility in the population, as well as high mortality since only 55% and 44% of children had living maternal and paternal grandmothers, respectively. Nonetheless, when grandmothers were present and provided care, they provided greater amounts of *high-*investment childcare (e.g. feeding the child or cleaning them), similar in type to maternal care. Overall, many children did not have access to a grandmother, and co-resident grandmothers did not necessarily help. However, children who were looked after by a grandmother received large amounts of high-investment childcare.

**Figure 1.**
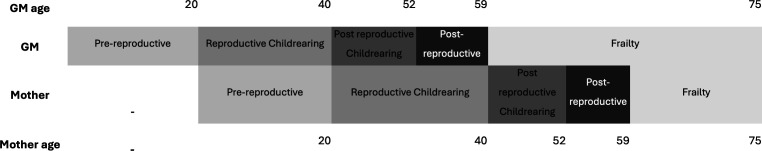
Life phase for grandmothers and mothers based on population averages. The ages at the top and the bottom represent the average age when this period finishes in the population, except for the ‘frailty’ age, which is from 59+ years. Each period is based on the average age of the last and first birth as well as life expectancy at age 45, however, in reality, different women move in and out of periods at slightly different times, in a more continuous fashion.

**Figure 2.**
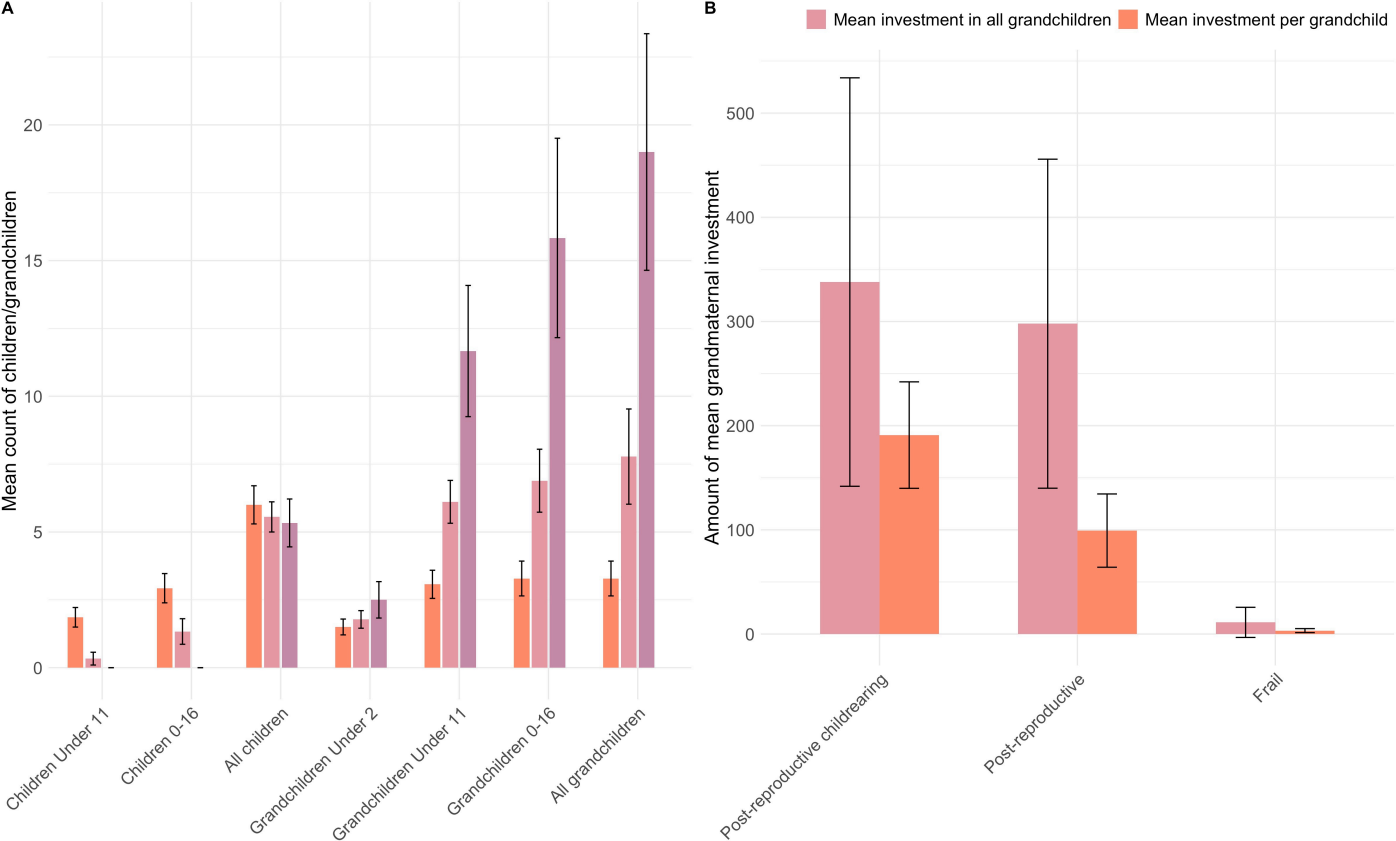
Number of children and grandchildren in the ages and mean investment in each grandchild, split by life phase of grandmothers (post-reproductive childrearing (40–52), grandmothering (52–59) and frailty/death (59+)).

**Figure 3.**
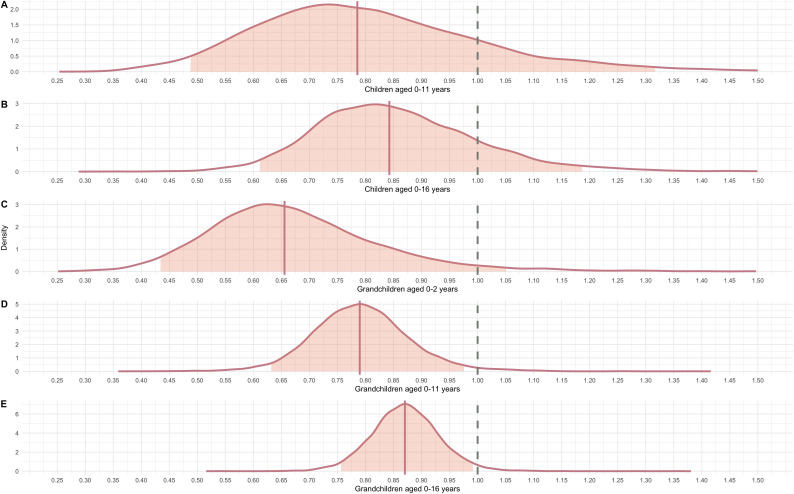
Posterior distributions for the five models predicting the count grandmaternal investment based on caregiving responsibilities: (A) children aged under 11 years, (B) children aged 0−16 years, (C) grandchildren aged 0−2 years, (D) grandchildren aged 0−11 years and (E) grandchildren aged 0−16 years. The dashed grey line represents the null, the solid line the modelled mean. The shaded zone is the 90% HPDI. The log odds are expressed as IRR, representing the rate of interactions between a grandmother and child.

**Figure 4.**
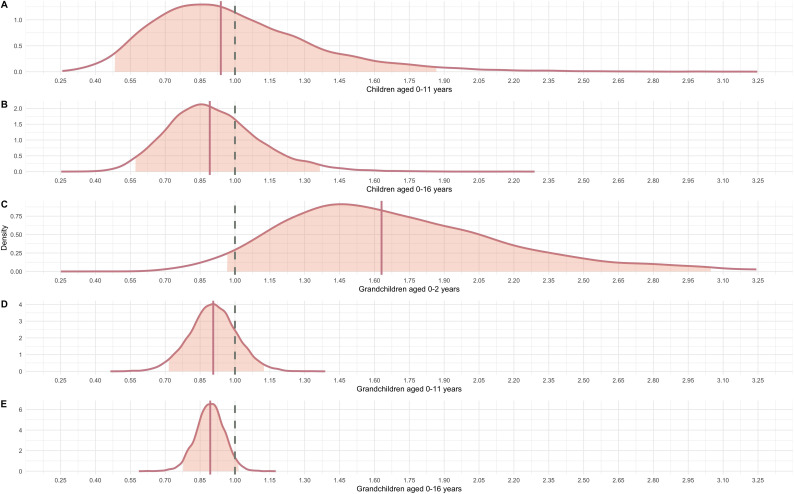
Posterior distributions for the five models predicting whether a grandmother invests at all for: (A) children aged under 11 years, (B) children aged 0−16 years, (C) grandchildren aged 0−2 years, (D) grandchildren aged 0−11 years and (E) grandchildren aged 0−16 years. The dashed grey line represents the null effect, the other line the modelled mean. The shaded zone is the 90% HPDI. The log odds are presented as odds ratios and represent the odds of grandmothers not interacting.

One factor underpinning grandmothering diversity is demography. In particular, expanding on Hill & Hurtado’s [9] point about co-residence and mortality to also include reproductive scheduling and total fertility. Reproductive schedules—specifically ages of first, last birth and the length of the interbirth interval—influence both (i) reproductive overlap between a grandmother and daughter/daughter-in-law [38–40] and (ii) competition between grandchildren [41], both of which are predicted to influence grandmaternal investment. While there is broad recognition that cooperation and competition are different sides of the same coin [42,43], the cooperative childrearing literature often (but not consistently, see [22,27,30]) assumes grandmothers to be helpful. Here, we detail the demographic schedules of the Agta with the aim of documenting the degree of this overlap and the likelihood of competition between and within generations. We then examine the relationship between the number of dependents a grandmother has with her investment in any one grandchild using Bayesian mixed-effect models, expecting that more dependents result in decreased investment.

## 2. Methods

### (a) The Agta

In 2013−14, there were around 1000 Agta living in the Palanan municipality of northeastern Luzon, Philippines. Riverine and marine spearfishing provides their primary source of animal protein, supplemented by hunting and gathering, as well as low-intensity cultivation, wage labour and trade [44,45]. This variation in subsistence is mirrored in the types of camps the Agta live in, as some camps are settled with permanent structures, are larger and have some form of infrastructure like a drinking well or church. Other camps are mobile, comprising temporary shelters, are smaller in size and the people who reside in these camps change frequently [46]. Previous research has highlighted their extensive cooperation between kin and non-kin across multiple domains from a young age [36,47–49]. The period total fertility rate ((TFR) the average number of children a woman would have over her lifetime based on the fertility rates observed in a specific period) in 2014 was 7.7, which was significantly higher among settled mothers (7.7) who engaged more in cultivation than mobile mothers who foraged more (6.6) [50]. Infant mortality was equally high, with 19% of children dying before their first year, 13.9% between 1 and 5 years, and 6% between 5 and 15 years [46].

### (b) Data collection

Data collection occurred over two field seasons from April to June 2013 and February to October 2014. This research was approved by the UCL Ethics Committee (UCL Ethics code 3086/003) and carried out with permission from the local government and representatives from the Agta community. Informed consent was obtained from all participants, and parents signed the informed consents for their children (after group and individual consultation and explanation of the research objectives in the indigenous language).

In the first season, we censused 915 Agta individuals (54.7% of which were male) across 20 camps. Following relative ageing protocols outlined in detail elsewhere [51], accurate ages were established for all individuals. Relatedness for the entire population was established from household genealogies alongside reproductive histories with all adult women encountered (*n* = 161), producing a record of all reported living (*n* = 632) and deceased (*n* = 210) children (see [52,53]).

In the second season, we stayed approximately 10−14 days in camps to conduct focal follows of children. Focal follows were conducted on 78 children (34 children aged 0−1.9; 44 children aged 2−5.9) in 10 study camps. No formal sampling techniques were used due to the small population size: we were able to observe the majority of children in our study camps (electronic supplementary material, table S1). Where complete focal follows on all children aged under 6 were not possible (e.g. in large camps), we observed at least one child per household. Although our total sample contains more boys than girls (48 males, 61.5%), this is in line with the male-biased sex ratio seen among children in this population [53]. Two researchers (A.E.P. and S.V.) observed each focal child for a 9 h period broken into three 4 h intervals (6.00–10.00, 10.00–14:00 and 14.00–18.00, with 15 min breaks each hour) on non-consecutive days. During observations, researchers recorded the activities of the focal child every 20 s, who else was within 3 m of the child and engaged in childcare (including carrying, holding, cleaning, playing and proximity watching and supervising). Further information can be found in the electronic supplementary material and [34].

### (c) Data analysis

Descriptive statistics were used to detail the key demographic features of the sample, which were used to create an ‘average Agta’ reproductive schedule. We then conducted Bayesian mixed-effect zero-inflated Poisson models to explore whether grandmothers’ dependency burden (the number of grandchildren and children she has) predicts the childcare investment made to any given grandchild. Zero-inflated Poisson models were used as the data include an excessive number of zeros—our data had 47/77 (61%) zeros as the outcome when grandmothers were alive but not present (not co-residing in the same camp), meeting this condition (electronic supplementary material, figure S5). In our data, there are two processes: (i) when a grandmother is present and helps by varying degrees (including not witnessed to help at all) and (ii) when a grandmother is not present and never able to help. This mixture model has two components to address these two processes. A count component via a Poisson model that explains the rate (incidence rate ratio, IRR) at which events occur when it can do so—the outcome being the number of interactions witnessed between a child and grandmother. The zero-inflated component models the probability of *excess* zeros occurring, via a logistic model predicting the odds (odds ratio, OR) of an observation being an extra zero rather than a count, representing when grandmothers were not present. We report and interpret both components of the model in the results.

All grandmother–grandchild dyads (*n* = 156) were entered into the dataset. If a grandmother was not present, but alive, this interaction was automatically recorded as a zero as they were not present to provide childcare. There were 47 zeros (30%) in the dataset, representing both when grandmothers were not present and present but provided no investment. If the grandmother was dead, the observations were recorded as missing and removed from analysis (*n* = 79 of 156, resulting in *n* = 77 observations). A mixed-effect structure was implemented to capture the non-independence of grandmothers (*n* = 29) as they had multiple grandchildren (*n* = 49).

We used directed acyclic graphs (DAGs) to illustrate the hypothesized causal relationships between variables and identify confounders to adjust for (see electronic supplementary material, figure S6) using the *dagitty* package [54]. Originally identified variables were: the number of children (aged 16 and under), number of grandchildren (aged 16 and under), grandmaternal age (continuous), settlement (binary, 1 = settled), the child’s age (continuous) and the sex of the child (binary, 1 = girl). Based on these causal assumptions, grandmaternal age (highlighted in orange) is a confounder, opening a backdoor between the number of dependent children/grandchildren (in purple) and the amount of childcare a grandmother gave (in green). Grandmaternal age is therefore controlled for in all models.

All models were run in R 3.2.2 [55] using the brms package [56], which fits Bayesian mixed-effect models. Across all models and components, weak normally distributed negative priors were set for the effects of dependents and grandmaternal age (mean = −0.25, s.d. = 0.5), as the small negative effect and broadness of the standard deviation allow the data to strongly influence the posterior estimate. Given convergence concerns with small sample sizes and the mixed-effect structure, the models were run over 20 000 iterations in 12 chains, a high delta (0.99, to ensure more accurate sampling) and a maximum tree depth of 30 (allowing longer paths to explore the posterior distribution more thoroughly given its complexity). All model diagnostics confirmed that the model fitting process was robust and reliable. This included verifying chain convergence (indicated by the R-hat values equal to 1), suggesting that the chains had converged to the target distribution. Additionally, the effective sample size (ESS) for each parameter was sufficiently large (minimal = 23 447), indicating a good representation of the posterior distribution. A visual inspection of trace plots further confirmed that the chains mixed well and sampled the posterior space adequately (electronic supplementary material, figures S7–S11). Divergent transitions were minimal to none, with no transitions exceeding the acceptable threshold, ensuring that the sampling algorithm explored the posterior space effectively without encountering problematic areas.

Overall, five separate models were run looking at the effect of number of dependents on grandmaternal childcare, separating out the predictors of (i) all children aged 11 and below, (ii) all children aged 16 and below, (iii) all grandchildren aged 2 and below, (iv) all grandchildren aged 11 and below, and (v) all grandchildren aged 16 and below. Age 0−2 was used as a measure of the most intensive period of mothering, when children are still breastfed on demand and yet to join the mixed-age, mixed-sex playgroups [34]. Ages 0−11 represent when children are still largely dependent on the care and provisioning of their parents. After this age, children’s productivity steadily increases as they become independent from their parents. Teenagers are also frequently absent from camps as they are free to explore and visit friends and relatives. As a result, after 11 years, children do not present high levels of investment demands on carers that limit investment elsewhere. By age 16, they become largely fully independent from their natal household, and start forming their own households [36,57]. We report 90% highest posterior density interval (HPDI) of posterior distributions from the multivariate models. We interpret associations between predictions and outcomes as strong when 90% of posterior distributions do not include 1 (e.g. posterior probability (PP) equal to, or higher than 0.9), moderate when 80% of posterior distributions do not include 1, and weak when less than 80% do not include 1 [50]. Full code and data required to replicate the analysis presented in this manuscript are available on the OSF project page: https://osf.io/t3e4w/.

## 3. Results

### (a) Demography of the population

Of 130 women interviewed (who had given birth), the mean age at first birth was 20.2 (s.d. = 4.3). The average age of the last birth for 37 post-reproductive women was 40.7 (s.d. = 5.9). Using data from post-reproductive individuals, the average fertile period was then 17.9 years (s.d. = 6.1) for women. The average inter-birth interval (IBI) was 2.7 years (s.d. = 1.4, *n* = 258). Since life tables are required to accurately assess age-dependent mortality rates, which we are unable to produce (due to difficulties producing age-specific rates over a short study period), our best estimate of life expectancy at the end of the reproductive period for women comes from Early & Headland’s overlapping sample of neighbouring Agta populations. They find life expectancy at age 45 is 14.0 years [58].

### (b) The helping window

Based on these averages, the life course of a woman can be separated into five distinct periods (figure 1). The first two represent the first 40 years of life: the ‘pre-reproductive phase’ (0 to 20 years) and the ‘reproductive childrearing phase’ (20 to 40 years). During this period, women’s childrearing responsibilities increase dramatically but are yet to have grandchildren (in our sample, the youngest grandmother was 38 years old). The next period is the ‘*post-reproductive childrearing phase’,* which is, on average, 11 years long (from age 41 to 52 years) after the cessation of live births, but when children remain dependent. At the same time, a woman will become a grandmother for the first time. Following this phase, women move into the fully ‘*post-reproductive phase’*, from 52 to 59 years, where they have limited responsibilities towards their own dependent children. This period ends at 59 years, reflective of the increased likelihood of ill health and death after this age. The final period is then the *‘frailty phase’* from 60 years onwards. This means that ‘the helping window’—when women have no more dependent children—for grandmothering is, on average, as short as 7 years.

### (c) Reproductive conflict and competition for cooperation

To explore the accuracy of our computed reproductive phases, we summarize grandmothers’ dependency burden and grandmaternal investment (count of caregiving observations for grandchildren aged under 6) by these periods (figure 2). Overall summary statistics are given in electronic supplementary material, table S2. No grandmothers had children aged 2 and below. Of the 29 grandmothers, 14 were aged under 52 years (‘*post-reproductive childrearing’*) and had on average 1.9 (s.d. = 1.4) children aged under 11 and 2.93 (s.d. = 2) children aged 0−16. They also had on average, 1.5 (s.d. = 1.1) grandchildren aged under 2 years, 3.1 (s.d. = 1.9) grandchildren under 11 and 3.3 (s.d. = 2.4) grandchildren under 16. Grandmothers in this category were observed to invest in any given grandchildren, on average, at 191 (s.d. = 245), of a possible (on average) 1082 observation points, representing 17.7%. Nine women were in the ‘*post-reproductive’* phase (age 52−59) and had, on average, 0.3 children aged under 11 years (s.d. = 0.7) and 1.3 (s.d. = 1.4) aged 0–16 years. They had 1.8 (s.d. = 1) grandchildren under 2 years, 6.1 (s.d. = 2.4) grandchildren under 11 and 6.9 (s.d. = 3.5) aged 0−16 years and 7.8 (s.d. = 5.3) living grandchildren. These individuals were observed to provide grandmaternal childcare, on average, on 99.3 (s.d. = 161.2, 9.2%) occasions, approximately half that of the childcare provided in the post-reproductive childrearing phase, per grandchild. Despite the decreased average investment *per grandchild* between the *post-reproductive childrearing* and *post-reproductive phases,* the total amount of grandmaternal care summed across all grandchildren per grandmother was similar (mean = 338, s.d. = 340 for *post-reproductive childrearing* versus mean = 298, s.d. = 273 for fully *post-reproductive,* figure 2B). This indicates that grandmothers in both phases invested similar amounts into direct childcare, post-reproductive grandmothers just had more grandchildren to divide this investment among.

The *‘frailty*’ phase included only six women, who had 0 children under 16 and 5.33 (s.d. = 2.2) living children, 2.5 (s.d. = 1.6) grandchildren aged 0−2 years, 11.7 (s.d. = 5.9) grandchildren aged 0−11 and 15.8 (s.d. = 9) grandchildren aged under 16. These women were only witnessed to interact with any given grandchildren on 2.39 occasions (s.d. = 7.9, 0.2%), on average, representing 1% and 3% of ‘*post-reproductive childrearing’* and ‘*post-reproductive’* phases, respectively. Grandmothers in the *frailty* phases also invested very little in childcare overall: the average total childcare provided was 11.2 (s.d. = 25, max = 56). This provides strong evidence that increasing grandmaternal age and frailty limit direct childcare from both the grandmother’s and grandchildren’s perspectives.

To summarize, given the Agta’s demographic schedules, ‘*post-reproductive childrearing’* grandmothers were in possible reproductive conflict with daughters/daughters-in-law as their caregiving burden remained high. Fully ‘*post-reproductive’* women had few children aged under 11, however, given high fertility rates, grandmothers had large numbers of grandchildren, creating competition for investment. The number of grandchildren continues to grow over time, which is the highest for the grandmothers in the ‘*frailty’* phase. Yet, all grandmothers had similar numbers of grandchildren under 2 (1.5−2.5), demonstrating their daughters/daughters-in-law were in high need at times when grandmothers had (i) their own young children (*post-reproductive childrearing*), (ii) large numbers of grandchildren of all ages (*post-reproductive* and *frailty* phases). Finally, given the low numbers of surviving grandmothers (in the ‘*frailty*’ phase’), and the very low to zero investment from those who did survive, older grandmothers are not a reliable source of childcare.

Based on these descriptive statistics, we developed testable hypotheses using our childcare observation data. Our first hypothesis is that grandmothers with more dependent children will invest less in each grandchild. Specifically, we predicted that (H1.1) more children will be associated with less grandmaternal childcare interactions with any given grandchild and (H1.2) that this effect (the frequency, the count component, and the likelihood of any interaction, the zero-inflated component) will be stronger for grandmothers with younger children. Our second hypothesis is that grandmothers with more grandchildren will invest less in each grandchild. Again, for the count component, we predicted that (H2.1) more grandchildren will be associated with less childcare interactions per grandchild and (H2.2) that this effect will be stronger for grandmothers with younger grandchildren. Given that the zero-inflated component captures the likelihood of any interaction, we had no prediction about the direction of the relationship between the number of grandchildren and the likelihood to be present.

### (d) Mixed-effect models for children and grandchildren: count results

Across the five models, predicting the effect of children (11 year and under and 0−16 years) and grandchildren (0−2 years, 0−11 years and 0−16 years), there is moderate to strong evidence that increasing dependency burden decreases investment in each grandchild, when controlling for grandmaternal age (figure 3). In the first model (figure 3A), for each additional child aged 11 or under, the rate of observed childcare interactions was estimated to decrease by 21.5% (IRR = 0.785, 90% HPDI [0.48, 1.32]). While this value is negative, there remains uncertainty since the credible interval is wide. Nonetheless, the PP (of the number of posterior distributions that do not include 0) is 0.84, representing moderate evidence. While model errors were high given the very small samples (indicating we should take predicted values with caution) based on this model, a grandmother with one child aged 0−11 years (estimated count = 82.9) is predicted to provide 21.7% more care to a grandchild than a grandmother with two children aged 0−11 years (estimated count = 68.1, full model estimates are available in electronic supplementary material, tables S3–S7 and predicted values in electronic supplementary material, table S8). A similar trend for children aged 0−16 years is apparent, while the effect size reduced slightly, in line with predictions (figure 3B). The rate of interactions decreases with each additional child aged 0−16 years by 15.8% (IRR = 0.84, 90% HPDI [0.61, 1.19], PP = 0.85), indicating moderate evidence for a negative effect. The model predicts that a grandmother with one child under 16 (estimated = 93.5) is expected to provide 17.9% more childcare to any given grandchild than a grandmother with two children (estimated = 79.3).

The last three models—of number of grandchildren aged 0−2, 0−11 and 0−16 years, respectively—provided strong evidence of a negative relationship between the count of grandchildren and childcare interactions, and the effects (as predicted) are stronger the younger the age of the grandchildren. In model 3 (figure 3C), one additional grandchild aged 0−2 years is associated with a 34.5% decrease in the rate of interactions (IRR = 0.66, 90% HDPI [0.43, 1.05], PP = 0.97). Based on this model, a grandmother with one infant grandchild (estimated amount = 123.1) is expected to provide approximately 50% more care to any given grandchild than a grandmother with two infant grandchildren (estimated amount = 79.4). The results for grandchildren aged 0−11 (IRR = 0.79, 90% HPDI [0.63, 0.98], PP = 0.98) and 0−16 (IRR = 0.87, 90% HPDI [0.75, 0.99], PP = 0.98) both similarly indicate negative relationships between observed childcare and number of dependent grandchildren, representing strong evidence. A grandmother with two grandchildren aged 0−11 is estimated to provide 414.4 instances of care, as compared to a grandmother with only one grandchild (aged 0−11), who is estimated to provide 587.6 instances of care. A grandmother with two grandchildren aged 0−16 (estimated amount = 346.3) is expected to provide 17.9% less care than a grandmother with only one grandchild (estimated amount = 418.7).

### (e) Mixed-effect models for children and grandchildren: zero-inflated results

The second component of our models seeks to understand what predicts the excess zeros—which are a measure of grandmothers providing no childcare at all. These results are shown in figure 4 and represent a less clear picture. Model 1 (children aged 0−11 years, figure 4A) is clearly a null finding (OR = 0.94, 90% HPDI [0.48, 1.89], PP = 0.57), alongside model 2 for children aged 0−16 years (OR = 0.89, 90% HPDI [0.57, 1.37], PP = 0.71, figure 4B). These models do not reliably capture variance in whether grandmothers provide any care or not.

In contrast, the results for model 3 (grandchildren aged 0−2, figure 4C) represent strong evidence of a large effect. With a PP of 0.96, there is a robust indication that the number of grandchildren aged 0−2 years is associated with the likelihood of grandmothers providing no care. Each additional grandchild aged 0−2 years was associated with an increased odds of 63.1% of a grandmother not providing any childcare (OR = 1.63, 90% HPDI [0.96, 3.1]). A grandmother with only one infant grandchild has a predicted probability of having zero interactions with any given grandchild of 0.59, as compared to 0.68 when they had five grandchildren aged 0−2 years, representing a 13.2% increase (full predicted probabilities are available in electronic supplementary material, table S9). The results for the number of grandchildren aged 0−11 and 0−16 show that more grandchildren of older ages are associated with a *decrease in odds* of grandmothers providing no childcare. The effects remain small, ranging from OR = 0.91 (90% HDPI [0.72, 1.13], PP = 0.83) for grandchildren 0−11 years to OR = 0.89 (90% HDPI [0.78, 1.02], *p* = 0.96) for grandchildren aged 0−16, but nonetheless provide strong evidence. Thus, it appears that while infant grandchildren are associated with an increase in odds of excess zeros, older grandchildren are associated with a decrease.

## 4. Discussion

Grandmothers being alive, present and physically able to help influences how much they do help, and these are arguably a function of demographic schedules. Similar to several other hunter–gatherer populations, previous studies have suggested that Agta children receive relatively little direct care from their grandmothers. Our results here suggest three simple but important demographic reasons for this. First, high levels of mortality in later life mean that many children do not have living grandmothers, and older grandmothers’ poorer health limits investment. Second, reproductive timing combined with a long period of childhood dependency means that there is an extended period in which grandmothers are still providing for their own children, reducing their investment in grandchildren by up to 21%. Third, high fertility rates mean that many women have a large number of grandchildren, diluting the amount of time they are able to invest in each grandchild by up to 34.5%. These findings are in line with our predictions developed from a comprehensive review of the Agta’s demographic schedules. The Agta, in a high fertility/mortality context—with extensive reproductive overlap, multiple competing grandchildren and high later life mortality—represent the most ‘restricted’ environment for grandmothering. This demographic perspective helps us understand diversity in grandmothering. Different populations—be that hunter–gatherer, horticulturalist, agriculturalists or large-scale populations—have different demographic schedules that may promote, or hinder, grandmothering. To test this, it is necessary to model the relationship between demography, grandmaternal availability and dependency burden, testing the predictions with large cross-cultural studies. Our exploratory case study in the Agta reveals the likely importance of demography, but future work is necessary to systematically understand demographic processes and unpick the diversity present in grandmothering worldwide.

### (a) Ageing and mortality

Age, frailty and mortality are important factors influencing grandmothering in the Agta. We see that 50% of children did not have living grandmothers due to high levels of female post-reproductive mortality, apparent in the extremely high sex ratios after the age of 55 [53]. These findings are in line with arguments made by Hill & Hurtado [9] that Ache and Hiwi grandmothers are unreliable sources of support, given high levels of mortality. The likelihood of grandmothers being alive is dependent on population-level trends in life expectancy, which change over time, allowing us to pull apart these effects to some degree. For instance, across the demographic transition in Finland (1790−1959), the percentage of children with living grandmothers increased from 36.0% and 43.6% in 1790 to 79.6% and 70.9% in the 1950s (for maternal and paternal grandmothers, respectively). The demographic transition, with its falls in mortality, can then increase the helping window of grandmothering [59]. However, as we have demonstrated, availability for grandmothering is a product of much more than just mortality [60].

Frailty is another key reason we see almost negligible levels of investment by grandmothers after the age of 60 when they are still alive. Likewise, in pre-industrial Finland, grandmothers faced an increase in mortality in their 60s, reaching a three times greater hazard of death by the age of 70 compared to 50. At the same time, grandmothers face the peak ‘grandmothering burden’ as multiple grandchildren are born. In this context, while younger children with grandmothers aged 50−75 years had an increased survivorship, this disappeared after the age of 75 due to large declines in grandmaternal health [39]. This health deterioration can also increase within-group resource competition as grandparents become more of a burden than a source of support [22]. This is evidenced by the fact that in pre-industrial Finland, having a living, but not co-resident, grandmother was the most beneficial for young infants, suggesting that co-residence may result in resource competition, shifting investment away from children [61]. It is evident then that one reason we see mixed results about the role of grandmothers [4,21,62] is related to the age of the grandmothers. Younger, more able grandmothers may provide more investment and take less resources. It is necessary, therefore, to understand this diversity in outcomes and roles to understand population-specific ageing and mortality.

### (b) Intergenerational conflict: children effect

Our results speak to the large literature on intergenerational conflict that occurs with generational reproductive overlap. One hypothesis for the evolution of the post-reproductive lifespan across species is that of the intergenerational harm caused by two generations simultaneously reproducing [63]. Specifically, within-group competition for resources is increased in a scenario where both the grandmother and mother reproduce, which will ultimately negatively impact the fitness of relatives. For instance, Mace & Alvergne [40] found in a rural Gambian population that when mothers and daughters reproduced at the same time, maternal grandmothers increased interbirth intervals and experienced a decreased probability of future births.

We have not explored the fitness consequences of reproductive overlap here, but it is evident that the helpfulness of grandmothers is dependent on their own fertility. The more children under the age of 16, and especially under the age of 11, the less they are invested in their grandchildren—arguably because the costs outweigh the benefits. Similar results were found in Swiss grandparents who decreased investment in grandchildren the more children they had, particularly when they started reproducing at younger ages [64]. Some lines of evidence have suggested that such intergenerational conflict is often won by the daughter due to age-linked relatedness asymmetries associated with female-biased dispersal [65]. Specifically, older women ceasing reproduction is favoured when women increase their relatedness to the local group as they age, increasing the benefits of cooperation, resulting in an extension of the non-reproductive lifespan to avoid intergenerational conflict [63,66]. In line with such models [65], if we are looking only at ages of first and last birth the Agta’s reproductive overlap and thus potential for conflict, is extremely low: old women stop reproducing (at 40) when their daughters start (at 20). However, here we have demonstrated the potential conflict stemming from childrearing overlap. In the Agta, we see significant overlap of childrearing: more children were clearly associated with reduced investment in grandchildren. In this case, older women were not forgoing their direct reproductive effort in favour of their daughters or daughters-in-law. One reason why we do not see the predictions based on age-linked relatedness asymmetries play out in the Agta is that, like many immediate-return hunter–gatherer populations, they have bilocal post-marriage residence systems, in which households frequently move locations [67,68]. There is no theoretical reason to expect relatedness to increase with age for women; the local group is too fluid. As a result, in the Agta, it appears that the specific reproductive schedule produces significant childrearing overlap; grandmothers are still mothers investing in their own children at the cost of their grandchildren.

It is noteworthy, however, that despite much focus on the negative impact of reproductive overlap, we find that ‘*post-reproductive childrearing’* grandmothers were investing slightly more into their entire group of grandchildren than the fully ‘*post-reproductive’* grandmothers. This suggests that other factors beyond intergenerational conflict are important in determining levels of grandmothering in the Agta.

### (c) Intragenerational conflict: grandchildren effect

Compared with intergenerational conflict, intragenerational conflict between siblings for parental care is less well considered in the context of grandmothering. Given the high levels of fertility in the Agta, grandmothers rapidly gained many grandchildren, and by the time they reached the ‘frailty’ age range had as many as 30 grandchildren. This, beyond declining health, is another reason why levels of investment decreased in any given grandchild. We have strong evidence that grandmothers with more grandchildren invested significantly less, when controlling for grandmaternal age. It follows that the more grandchildren a grandmother has—all else being equal—the more her care may be diluted, as demonstrated elsewhere [41,64,69]. In the Agta, due to reproductive overlap, mothers are not able to rely on their mother, or mother-in-law, to be present and able to help. This may be why we see—in similar hunter–gatherer contexts—wide allomothering networks of diverse combinations of kin and non-kin [3,36].

Multiple grandchildren hint at competition between families for grandmaternal care, especially if grandmaternal care is an important means of ensuring child survival. In line with the wider cooperation literature, grandmothers may be investing strategically [49,70,71]. Specifically, inclusive fitness returns of any given investment are increased when a mother–child dyad most needs it because this increases the benefits relative to the costs [36]. This may be why we see increased investment in younger grandchildren because mothers’ time and productivity are most constrained when they have young children [72,73]. As documented in the Agta and elsewhere, grandmothers are documented to do more high investment, targeted help to younger grandchildren in the form of feeding, washing and providing medical help [34,74]. From this perspective, our results that grandmothers with grandchildren aged under 2 years are less likely to be present for any given grandchild make sense. Agta grandmothers may target their investment to mothers with younger children, investing a lot in that one grandchild to the exclusion of others—all is not equal, and care is not uniformly diluted among large grandchildren sets. Notably, from the grandmother’s perspective, ‘*childrearing post-reproductive’* and ‘*post-reproductive*’ grandmothers invested at similar overall levels, just divided between more grandchildren as they aged, reducing the average. As a result, while the grandmothering effect appears diluted from the children’s perspective, targeted investment in a singular grandchild may be sufficient to produce an overall fitness benefit. Certainly, Chapman *et al*. [59] make this argument that even when grandmothers are rarely available or the overlap between generations short, if the fitness benefits are large enough and targeted at needy ages, then grandmothering remains adaptive. Arguably, however, the fitness consequences of grandmaternal availability on investment (given their dependences on both fertility and mortality) in either multiple or singular ‘needy’ offspring is complex to capture in verbal models. Future work must formally model these dynamics to fully test the grandmothering hypothesis.

We also found that more grandchildren decreased the odds of excess zeros in our dataset, indicating that more grandchildren meant grandmothers were *more likely* to be co-resident. A possible interpretation here is related to strategic co-residence, where grandmothers with older grandchildren aged 2 and above may co-reside with families with lots of children. Families with many older children—not uncommon in a population with a high TFR—may represent a different type of need where grandmothers can provide more low-investment supervision and proximity childcare [36]. This approach would be especially effective since similarly aged adult siblings often co-reside in the same camp, meaning grandmothers would have access to several sibling sets. In such a situation, grandmothers are able to invest low levels into many of their grandchildren, reconciling our findings that more grandchildren decreased investment in each grandchild, but increases the likelihood of co-residence (proxied by reduction in excess zeros). Koster *et al*. [75] note a similar trend in the Mayangna and Maya due to a bottom-heavy population pyramid, older grandmothers with many grandchildren tended to co-reside. In such cases, investing in collective goods to large groups of grandchildren may be a more effective investment strategy overall.

### (d) Demography of grandmothering

Thinking about grandmothering in a demographic light helps us understand *why* we see such diversity in grandmother roles across studies and societies [76]. It is evident across populations that there is little consistency in both levels of grandmaternal help and the consequences of this help [4,21,26]. Our findings demonstrate that grandmaternal investment is dependent on both generations’ reproductive timing and total fertility in line with the demographic literature [77], directly impacting the likelihood that grandmothers are alive, present and able to help. The Agta, as a high fertility/mortality population, represent perhaps a very ‘restricted’ case for grandmothering, unlike what is present in many high-income populations today [78]. However, the Agta case is not representative of all hunter–gatherer groups, given the demography of these populations varies significantly [50,57,79,80]. Across hunter–gatherers, grandmaternal care appears to be the *most* variable, ranging from 2% to 21% of observed allomothering [33]. For instance, grandmothers from the BaYaka hunter–gatherers (Congo) contribute four times more to direct caregiving compared with the Agta. This appears to be the product of differential fertility schedules: age-specific fertility is higher at older ages in the Agta, increasing reproductive overlap and the Agta have significantly higher TFR, increasing competition of grandmaternal resources [33]. Furthermore, the Ache who have very low levels of grandmaternal investment, also have very late ages of last reproduction, increasing reproductive overlap [9,81]. Clearly, demography is important, but how do we systematically explore the role of demography on grandmothering to help us better unpick the diversity across and within populations?

Within formal demography, especially kinship demography, methodological approaches are being applied to systematically explore the demography of grandmothering and kinship overlap. Since ‘informal’ caregiving like grandmothering is dependent on complex generational processes, microsimulation and matrix models are necessary to analyse how the relationships between mortality and fertility play out between generations [82,83]. Specifically, verbal models are less useful in these contexts because of the need to account for the dependent and non-intuitive effects of fertility and mortality. For instance, in a study of how grandparenting changed in Canada over a 26 year period, extended households became more common as survivorship increased over time, however, low fertility reduced the proportion of the population who ever became grandmothers [78]. Further, the availability of grandmothers can vary due to slight demographic changes in very similar contexts. For instance, in eastern Germany, grandparents frequently were yet to retire given much earlier ages of first reproduction as compared to western Germany [77], increasing conflict with ‘working roles’. Even in large-scale national states, there is significant demographic variations, which mean the ‘happy retired grandmother’ is far more likely in some contexts (Western Europe) than others (the US and Eastern Europe) [60]. As a result, to gain a better understanding of the evolution of human cooperative reproduction, and particularly variation in the role of grandmothers, formal models are required in pre-demographic transition contexts in which fertility and mortality are both high and variable [84].

### (e) Limitations

Our analysis suffers a number of limitations that are common, and difficult to avoid, in anthropological studies of childcare in small-scale communities. Firstly, our sample sizes are small. This study is based on observations of 78 children of whom 47 could have received care from 29 grandmothers. This is reflective of the small population size overall (roughly 1000 individuals), the high mortality rate of older women in the population and the resource and time-consuming nature of such granular data. For this reason, we implemented Bayesian models to overcome the limitations of a frequentist framework with small samples, but nonetheless, much of the uncertainty (large credible intervals) in our models likely stems from this. Equally, here we only analyse one form of allomothering—direct childcare. Allomothering also comes in the form of provisioning of resources [9,10], conducting domestic tasks [17] and informational and emotional support [1,7]. It is not possible to be confident about the entirety of grandmother’s investment until cooperation across domains has been captured [85]. This is particularly important if one reason why grandmothers are documented to be important in the Hadza, as compared to South American foragers, is related to the foraging environment and if knowledge and skills become more important over time, rather than strength. Finally, while our use of DAGs supports the causal interpretation of the results, a potential limitation arises from unmeasured confounding variables, such as the health or fitness level of grandmothers. For instance, a healthier grandmother might be capable of having more children and grandchildren and also be more inclined or able to provide help, thereby introducing bias into the analysis due to these unobserved factors.

## 5. Conclusion

The findings from this study underscore the significant role that demographic factors play in shaping grandmaternal involvement in childcare among the Agta foragers. High mortality rates, reproductive overlap and the extensive burden of multiple grandchildren collectively constrain the ability of grandmothers to provide consistent childcare. Specifically, increasing age, numbers of children and grandchildren decrease grandmaternal investment. These results highlight why grandmothering is not universally prevalent or beneficial but is deeply influenced by demographic schedules. Future research utilizing cross-cultural and formal demographic models is essential to better understand the variability and evolutionary significance of grandmothering in human societies.

## Data Availability

Full code and data required to replicate the analysis presented in this manuscript is available on the OSF project page [86]. Supplementary material is available online [87].

## References

[1] Emmott EH, Page AE. 2019 Alloparenting. In Encyclopedia of evolutionary psychological science (eds TK Shackelford, VA Weekes-Shackelford), pp. 1–14. Cham, Switzerland: Springer International Publishing. (10.1007/978-3-319-16999-6_2253-1)

[2] Hassan A, Lawson DW, Schaffnit SB, Urassa M, Sear R. 2021 Childcare in transition: evidence that patterns of childcare differ by degree of market integration in north-western Tanzania. OSF Preprints. (10.31219/osf.io/gtc6k) 42627165

[3] Helfrecht C, Roulette JW, Lane A, Sintayehu B, Meehan CL. 2020 Life history and socioecology of infancy. Am. J. Phys. Anthropol. **173**, 619–629. (10.1002/ajpa.24145) 32955732

[4] Kramer KL. 2010 Cooperative breeding and its significance to the demographic success of humans. Annu. Rev. Anthropol. **39**, 417–436. (10.1146/annurev.anthro.012809.105054)

[5] Sear R, Mace R. 2008 Who keeps children alive? A review of the effects of kin on child survival. Evol. Hum. Behav. **29**, 1–18. (10.1016/j.evolhumbehav.2007.10.001)

[6] Valeggia CR. 2009 Flexible caretakers: responses of Toba families in transition. In Substitute parents: biological and social perspective on alloparenting across human societies (eds GR Bentley, R Mace), pp. 100–114. New York, NY: Berghahn Books. (10.1515/9781845459536-008)

[7] Scelza BA, Hinde K. 2019 Crucial contributions. Hum. Nat. **30**, 371–397. (10.1007/s12110-019-09356-2) 31802396 PMC6911617

[8] Myers S, Page AE, Emmott EH. 2021 The differential role of practical and emotional support in infant feeding experience in the UK. Phil. Trans. R. Soc. B **376**, 20200034. (10.1098/rstb.2020.0034) 33938282 PMC8090825

[9] Hill K, Hurtado AM. 2009 Cooperative breeding in South American hunter–gatherers. Proc. R. Soc. B **276**, 3863–3870. (10.1098/rspb.2009.1061) PMC2817285 19692401

[10] Hawkes K, O’Connell JF, Blurton Jones NG. 1997 Hadza women’s time allocation, offspring provisioning, and the evolution of long postmenopausal life spans. Curr. Anthropol. **38**, 551–577. (10.1086/204646)

[11] Hrdy SB. 2005 Cooperative breeders with an ace in the hole. In Grandmotherhood: the evolutionary significance of the second half of the female life (eds E Voland, A Chasiotis), pp. 295–317. New Brunswick, NJ: Rutgers University Press.

[12] Adams AM, Madhavan S, Simon D. 2002 Women’s social networks and child survival in Mali. Soc. Sci. Med. **54**, 165–178. (10.1016/s0277-9536(01)00017-x) 11824923

[13] Aubel J. 2012 The role and influence of grandmothers on child nutrition: culturally designated advisors and caregivers. Matern. Child Nutr. **8**, 19–35. (10.1111/j.1740-8709.2011.00333.x) 21951995 PMC6860857

[14] Beise J. 2005 The helping grandmother and the helpful grandmother: The role of maternal and paternal grandmothers in child mortality in the 17th and 18th century population of French settlers in Quebec, Canada. In Grandmotherhood: the evolutionary significance of the second half of the female life (eds E Voland, A Chasiotis, W Schiefenhovel), pp. 215–238. New Brunswick, NJ: Rutgers University Press.

[15] Chapman SN, Lahdenperä M, Pettay JE, Lynch RF, Lummaa V. 2021 Offspring fertility and grandchild survival enhanced by maternal grandmothers in a pre-industrial human society. Sci. Rep. **11**, 11. (10.1038/s41598-021-83353-3) 33574488 PMC7878921

[16] Cunningham SA, Elo IT, Herbst K, Hosegood V. 2010 Prenatal development in rural South Africa: relationship between birth weight and access to fathers and grandparents. Popul. Stud. **64**, 229–246. (10.1080/00324728.2010.510201) PMC2988436 20954098

[17] Gibson MA, Mace R. 2005 Helpful grandmothers in rural Ethiopia: a study of the effect of kin on child survival and growth. Evol. Hum. Behav. **26**, 469–482. (10.1016/j.evolhumbehav.2005.03.004)

[18] Karimli L, Ssewamala FM, Ismayilova L. 2012 Extended families and perceived caregiver support to AIDS orphans in Rakai district of Uganda. Child. Youth Serv. Rev. **34**, 1351–1358. (10.1016/j.childyouth.2012.03.015) 23188930 PMC3505487

[19] Madhavan S. 2010 Early childbearing and kin connectivity in rural South Africa. Int. J. Sociol. Fam **36**, 139–157. (10.2307/23028826)

[20] Meehan CL, Quinlan R, Malcom CD. 2013 Cooperative breeding and maternal energy expenditure among aka foragers. Am. J. Hum. Biol. **25**, 42–57. (10.1002/ajhb.22336) 23203600

[21] Sear R, Coall D. 2011 How much does family matter? Cooperative breeding and the demographic transition. Popul. Dev. Rev. **37**, 81–112. (10.1111/j.1728-4457.2011.00379.x) 21280366

[22] Strassmann BI, Garrard WM. 2011 Alternatives to the grandmother hypothesis. Hum. Nat. **22**, 201–222. (10.1007/s12110-011-9114-8) 22388808

[23] Hawkes K, O’Connell JF, Blurton Jones NG. 2003 Human life histories: primate trade-offs, grandmothering, socioecology and the fossil record. In Primate life histories and socioecology (eds PM Kappeler, ME Pereira), pp. 204–232. London, UK: The University of Chicago Press.

[24] Hawkes K, Coxworth JE. 2013 Grandmothers and the evolution of human longevity: a review of findings and future directions. Evol. Anthropol. **22**, 294–302. (10.1002/evan.21382) 24347503

[25] Lahdenperä M, Lummaa V, Helle S, Tremblay M, Russell AF. 2004 Fitness benefits of prolonged post-reproductive lifespan in women. Nature **428**, 178–181. (10.1038/nature02367) 15014499

[26] Sadruddin AFA, Ponguta LA, Zonderman AL, Wiley KS, Grimshaw A, Panter-Brick C. 2019 How do grandparents influence child health and development? A systematic review. Soc. Sci. Med. **239**, 112476. (10.1016/j.socscimed.2019.112476) 31539783

[27] Sear R. 2008 Kin and child survival in rural Malawi. Hum. Nat. **19**, 277–293. (10.1007/s12110-008-9042-4) 26181618

[28] Beise J, Voland E. 2002 A multilevel event history analysis of the effects of grandmothers on child mortality in a historical German population. Demogr. Res. **7**, 469–498. (10.4054/DemRes.2002.7.13)

[29] Jamison CS, Cornell LL, Jamison PL, Nakazato H. 2002 Are all grandmothers equal? A review and a preliminary test of the ‘grandmother hypothesis’ in Tokugawa Japan. Am. J. Phys. Anthropol. **119**, 67–76. (10.1002/ajpa.10070) 12209574

[30] Sheppard P, Sear R. 2016 Do grandparents compete with or support their grandchildren? R. Soc. Open. Sci. **3**, 160069. (10.1098/rsos.160069) 27152221 PMC4852644

[31] Crittenden AN, Marlowe FW. 2008 Allomaternal care among the Hadza of Tanzania. Hum. Nat. **19**, 249–262. (10.1007/s12110-008-9043-3) 26181616

[32] Kramer KL. 2005 Children’s help and the pace of reproduction: cooperative breeding in humans. Evol. Anthropol. **14**, 224–237. (10.1002/evan.20082)

[33] Chaudhary N, Page AE, Salali GD, Dyble M, Major-Smith D, Migliano AB, Vinicius L, Thompson J, Viguier S. 2024 Hunter–gatherer children’s close-proximity networks: similarities and differences with cooperative and communal breeding systems. Evol. Hum. Sci. **6**, e11. (10.1017/ehs.2024.1) 38516373 PMC10955362

[34] Page AE, Emmott EH, Dyble M, Smith D, Chaudhary N, Viguier S, Migliano AB. 2021 Children are important too: juvenile playgroups and maternal childcare in a foraging population, the Agta. Phil. Trans. R. Soc. B **376**, 20200026. (10.1098/rstb.2020.0026) 33938270 PMC8090817

[35] Page AE, Migliano AB, Dyble M, Major-Smith D, Viguier S, Hassan A. 2023 Sedentarization and maternal childcare networks: role of risk, gender and demography. Phil. Trans. R. Soc. B **378**, 20210435. (10.1098/rstb.2021.0435) 36440566 PMC9703224

[36] Page AE *et al*. 2019 Testing adaptive hypotheses of alloparenting in Agta foragers. Nat. Hum. Behav. **3**, 1154–1163. (10.1038/s41562-019-0679-2) 31406338 PMC6858278

[37] Chaudhary N, Salali GD, Swanepoel A. 2024 Sensitive responsiveness and multiple caregiving networks among Mbendjele BaYaka hunter–gatherers: potential implications for psychological development and well-being. Dev. Psychol. **60**, 422–440. (10.1037/dev0001601) 37956035

[38] Lahdenperä M, Gillespie DOS, Lummaa V, Russell AF. 2012 Severe intergenerational reproductive conflict and the evolution of menopause. Ecol. Lett. **15**, 1283–1290. (10.1111/j.1461-0248.2012.01851.x) 22913671

[39] Chapman SN, Pettay JE, Lummaa V, Lahdenperä M. 2019 Limits to fitness benefits of prolonged post-reproductive lifespan in women. Curr. Biol. **29**, 645–650.(10.1016/j.cub.2018.12.052) 30744967

[40] Mace R, Alvergne A. 2012 Female reproductive competition within families in rural Gambia. Proc. R. Soc. B **279**, 2219–2227. (10.1098/rspb.2011.2424) PMC3321706 22258635

[41] Euler HA. 2011 Grandparents and extended kin. In The Oxford handbook of evolutionary family psychology (eds CA Salmon, TK Shackelford), pp. 181–210. Oxford, UK: Oxford University Press. (10.5860/CHOICE.49-2372)

[42] Moya C, Sear R. 2014 Intergenerational conflicts may help explain parental absence effects on reproductive timing: a model of age at first birth in humans. PeerJ **2**, e512. (10.7717/peerj.512) 25165627 PMC4137655

[43] Du J, Huang Y, Bai PP, Zhou L, Myers S, Page AE, Mace R. 2023 Post-marital residence patterns and the timing of reproduction: evidence from a matrilineal society. Proc. R. Soc. B **290**, 20230159. (10.1098/rspb.2023.0159) PMC10031416 36946117

[44] Minter T. 2010 The Agta of the northern Sierra Madre: livelihood strategies and resilience among Philippine hunter–gatherers. PhD thesis, Leiden University, Leiden, The Netherlands.

[45] Dyble M, Thorley J, Page AE, Smith D, Migliano AB. 2019 Engagement in agricultural work is associated with reduced leisure time among Agta hunter–gatherers. Nat. Hum. Behav. **3**, 792–796. (10.1038/s41562-019-0614-6) 31110340

[46] Page AE *et al*. 2016 Reproductive trade-offs in extant hunter–gatherers suggest adaptive mechanism for the Neolithic expansion. Proc. Natl Acad. Sci. USA **113**, 4694–4699. (10.1073/pnas.1524031113) 27071109 PMC4855554

[47] Dyble M, Thompson J, Smith D, Salali GD, Chaudhary N, Page AE, Vinicuis L, Mace R, Migliano AB. 2016 Networks of food sharing reveal the functional significance of multilevel sociality in two hunter–gatherer groups. Curr. Biol. **26**, 2017–2021. (10.1016/j.cub.2016.05.064) 27451900

[48] Major-Smith D, Chaudhary N, Dyble M, Major-Smith K, Page AE, Salali GD, Mace R, Migliano AB. 2023 Cooperation and partner choice among Agta hunter–gatherer children: an evolutionary developmental perspective. PLoS One **18**, e0284360. (10.1371/journal.pone.0284360) 37099506 PMC10132543

[49] Smith D *et al*. 2019 A friend in need is a friend indeed: need-based sharing, rather than cooperative assortment, predicts experimental resource transfers among Agta hunter–gatherers. Evol. Hum. Behav. **40**, 82–89. (10.1016/j.evolhumbehav.2018.08.004)

[50] Page AE *et al*. 2024 Women’s subsistence strategies predict fertility across cultures, but context matters. Proc. Natl Acad. Sci. USA **121**, e2318181121. (10.1073/pnas.2318181121) 38346210 PMC10907265

[51] Diekmann Y, Smith D, Gerbault P, Dyble M, Page AE, Chaudhary N, Migliano AB, Thomas MG. 2017 Accurate age estimation in small-scale societies. Proc. Natl Acad. Sci. USA **114**, 8205–8210. (10.1073/pnas.1619583114) 28696282 PMC5547587

[52] Dyble M, Salali GD, Chaudhary N, Page AE, Smith D, Thompson J, Vinicius L, Mace R, Migliano AB. 2015 Sex equality can explain the unique social structure of hunter–gatherer bands. Science **348**, 796–798. (10.1126/science.aaa5139) 25977551

[53] Page AE, Myers S, Dyble M, Migliano AB. 2019 Why so many Agta boys? Explaining ‘extreme’ sex ratios in Philippine foragers. Evol. Hum. Sci. **1**, e5. (10.1017/ehs.2019.4) 37588404 PMC10427305

[54] Textor J, van der Zander B, Gilthorpe MS, Liśkiewicz M, Ellison GTH. 2017 Robust causal inference using directed acyclic graphs: the R package ‘dagitty’. Int. J. Epidemiol. **45**, 1887–1894. (10.1093/ije/dyw341) 28089956

[55] R Core Team. 2021 R: a language and environment for statistical computing. Vienna, Austria: R Foundation for Statistical Computing.

[56] Bürkner PC. 2017 brms: An R package for Bayesian multilevel models using Stan. J. Stat. Softw. **80**, 01. (10.18637/jss.v080.i01)

[57] Page AE, French JC. 2020 Reconstructing prehistoric demography: what role for extant hunter–gatherers? Evol. Anthropol. Issues News Rev. **29**, 332–345. (10.1002/evan.21869) 33103830

[58] Early JD, Headland TN. 1998 Population dynamics of a Philippine rain forest people: the San ildefonso Agta. Gainesville, FL, USA: University Press of Florida.

[59] Chapman SN, Pettay JE, Lahdenperä M, Lummaa V. 2018 Grandmotherhood across the demographic transition. PLoS One **13**, e0200963. (10.1371/journal.pone.0200963) 30036378 PMC6056041

[60] Leopold T, Skopek J. 2015 The demography of grandparenthood: an international profile. Soc. Forces **94**, 801–832. (10.1093/sf/sov066)

[61] Chapman S, Danielsbacka M, Tanskanen AO, Lahdenperä M, Pettay J, Lummaa V. 2023 Grandparental co-residence and grandchild survival: the role of resource competition in a pre-industrial population. Behav. Ecol. **34**, 446–456. (10.1093/beheco/arad013) 37192925 PMC10183204

[62] Tanskanen AO, Danielsbacka M. 2018 Multigenerational effects on children’s cognitive and socioemotional outcomes: a within‐child investigation. Child Dev. **89**, 1856–1870. (10.1111/cdev.12968) 28960255

[63] Ellis S, Franks DW, Nielsen MLK, Weiss MN, Croft DP. 2024 The evolution of menopause in toothed whales. Nature **627**, 579–585. (10.1038/s41586-024-07159-9) 38480878 PMC10954554

[64] Coall DA, Meier M, Hertwig R, Wänke M, Höpflinger F. 2009 Grandparental investment: the influence of reproductive timing and family size. Am. J. Hum. Biol. **21**, 455–463. (10.1002/ajhb.20894) 19298005

[65] Cant MA, Johnstone RA. 2008 Reproductive conflict and the separation of reproductive generations in humans. Proc. Natl Acad. Sci. USA **105**, 5332–5336. (10.1073/pnas.0711911105) 18378891 PMC2291103

[66] Croft DP *et al*. 2017 Reproductive conflict and the evolution of menopause in killer whales. Curr. Biol. **27**, 298–304. (10.1016/j.cub.2016.12.015) 28089514

[67] Hassan A, Page AE. 2025 Family types and sizes. SocArXiv. (10.31235/osf.io/5wrcu)

[68] Marlowe FW. 2004 Marital residence among foragers. Curr. Anthropol. **45**, 277–284. (10.1086/382256)

[69] Uhlenberg P, Hammill BG. 1998 Frequency of grandparent contact with grandchild sets: six factors that make a difference. Gerontol. **38**, 276–285. (10.1093/geront/38.3.276) 9640847

[70] Hao Y, Armbruster D, Cronk L, Aktipis CA. 2015 Need-based transfers on a network: a model of risk-pooling in ecologically volatile environments. Evol. Hum. Behav. **36**, 265–273. (10.1016/j.evolhumbehav.2014.12.003)

[71] Snopkowski K, Sear R. 2015 Grandparental help in Indonesia is directed preferentially towards needier descendants: a potential confounder when exploring grandparental influences on child health. Soc. Sci. Med. **128**, 105–114. (10.1016/j.socscimed.2015.01.012) 25603472

[72] Kaplan H, Hill K, Lancaster J, Hurtado AM. 2000 A theory of human life history evolution: diet, intelligence, and longevity. Evol. Anthropol. **9**, 156–185. (10.1002/1520-6505(2000)9:43.3.co;2-z)

[73] Lee RD, Kramer KL. 2002 Children’s economic roles in the Maya family life cycle: Cain, Caldwell, and Chayanov revisited. Popul. Dev. Rev. **28**, 475–499. (10.1111/j.1728-4457.2002.00475.x)

[74] Scelza BA. 2009 The grandmaternal niche: critical caretaking among Martu Aborigines. Am. J. Hum. Biol. **21**, 448–454. (10.1002/ajhb.20934) 19402034

[75] Koster J *et al*. 2019 Kinship ties across the lifespan in human communities. Phil. Trans. R. Soc. B **374**, 20180069. (10.1098/rstb.2018.0069) 31303163 PMC6664140

[76] Chapman SN, Lummaa V. 2024 Grandmother effects over the Finnish demographic transition. Evol. Hum. Sci. **6**, 1–19. (10.1017/ehs.2023.36) PMC10955376 38516365

[77] Leopold T, Skopek J. 2015 The delay of grandparenthood: a cohort comparison in East and West Germany. J. Marriage Fam. **77**, 441–460. (10.1111/jomf.12169)

[78] Margolis R. 2016 The changing demography of grandparenthood. J. Marriage Fam. **78**, 610–622. (10.1111/jomf.12286)

[79] Campbell KL, Wood JW. 1988 Fertility in traditional societies. In Natural human fertility (eds P Diggory, S Teper, M Potts), pp. 39–69. London, UK: Macmillan Publishing. (10.1007/978-1-349-09961-0_4)

[80] Hewlett BS. 1991 Demography and childcare in preindustrial societies. J. Anthropol. Res. **42**, 1–37. (10.1086/jar.47.1.3630579) 12317265

[81] Migliano AB, Vinicius L, Lahr MM. 2007 Life history trade-offs explain the evolution of human pygmies. Proc. Natl Acad. Sci. USA **104**, 20216–20219. (10.1073/pnas.0708024105) 18077366 PMC2154411

[82] Alburez‐Gutierrez D, Mason C, Zagheni E. 2021 The ‘Sandwich Generation’ Revisited: Global Demographic Drivers of Care Time Demands. Popul. Dev. Rev. **47**, 997–1023. (10.1111/padr.12436)

[83] Caswell H, Vries C. 2025 The formal demography of kinship. VII. Lifetime kin overlap within and across generations. bioRxiv 2025.03.05.641714. (10.1101/2025.03.05.641714)

[84] Kramer KL, Hackman J, Schacht R, Davis HE. 2021 Effects of family planning on fertility behaviour across the demographic transition. Sci. Rep. **11**, 8835. (10.1038/s41598-021-86180-8) 33893324 PMC8065026

[85] Jaeggi AV, Hooper PL, Beheim BA, Kaplan H, Gurven M. 2016 Reciprocal exchange patterned by market forces helps explain cooperation in a small-scale society. Curr. Biol. **26**, 2180–2187. (10.1016/j.cub.2016.06.019) 27451903

[86] Page AE, Dyble M, Migliano A, Chaudhary N, Viguier S, Major-Smith D. 2025 Demography of grandmothering—a case study in Agta foragers. OSF. See https://osf.io/t3e4w/.10.1098/rspb.2025.038540359976

[87] Page AE, Dyble M, Migliano A, Chaudhary N, Viguier S, Major-Smith D. 2025 Supplementary material from: Demography of grandmothering: a case study in Agta foragers. Figshare (10.6084/m9.figshare.c.7802074)40359976

